# Long branch-chains of amylopectin with B-type crystallinity in rice seed with inhibition of starch branching enzyme I and IIb resist in situ degradation and inhibit plant growth during seedling development: Degradation of rice starch with inhibition of SBEI/IIb during seedling development

**DOI:** 10.1186/s12870-017-1219-8

**Published:** 2018-01-08

**Authors:** Ting Pan, Lingshang Lin, Juan Wang, Qiaoquan Liu, Cunxu Wei

**Affiliations:** 1 Key Laboratory of Crop Genetics and Physiology of Jiangsu Province/Key Laboratory of Plant Functional Genomics of the Ministry of Education, Yangzhou University, Yangzhou, 225009 China; 2 Co-Innovation Center for Modern Production Technology of Grain Crops of Jiangsu Province/Joint International Research Laboratory of Agriculture & Agri-Product Safety of the Ministry of Education, Yangzhou University, Yangzhou, 225009 China

**Keywords:** Rice, Resistant starch, In situ degradation, Seedling growth, Starch properties

## Abstract

**Background:**

Endosperm starch provides prime energy for cereal seedling growth. Cereal endosperm with repression of starch branching enzyme (SBE) has been widely studied for its high resistant starch content and health benefit. However, in barley and maize, the repression of SBE changes starch component and amylopectin structure which affects grain germination and seedling establishment. A high resistant starch rice line (TRS) has been developed through inhibiting SBEI/IIb, and its starch has very high resistance to in vitro hydrolysis and digestion. However, it is unclear whether the starch resists in situ degradation in seed and influences seedling growth after grain germination.

**Results:**

In this study, TRS and its wild-type rice cultivar Te-qing (TQ) were used to investigate the seedling growth, starch property changes, and in situ starch degradation during seedling growth. The slow degradation of starch in TRS seed restrained the seedling growth. The starch components including amylose and amylopectin were simultaneously degraded in TQ seeds during seedling growth, but in TRS seeds, the amylose was degraded faster than amylopectin and the amylopectin long branch-chains with B-type crystallinity had high resistance to in situ degradation. TQ starch was gradually degraded from the proximal to distal region of embryo and from the outer to inner in endosperm. However, TRS endosperm contained polygonal, aggregate, elongated and hollow starch from inner to outer. The polygonal starch similar to TQ starch was completely degraded, and the other starches with long branch-chains of amylopectin and B-type crystallinity were degraded faster at the early stage of seedling growth but had high resistance to in situ degradation during TRS seedling growth.

**Conclusions:**

The B-type crystallinity and long branch-chains of amylopectin in TRS seed had high resistance to in situ degradation, which inhibited TRS seedling growth.

**Electronic supplementary material:**

The online version of this article (10.1186/s12870-017-1219-8) contains supplementary material, which is available to authorized users.

## Background

Cereal endosperm starch is a major source of nourishment for humans, and consists of two main components: linear amylose and highly branched amylopectin. Amylose content and amylopectin structure have important effects on structural and functional properties of starch. The amylose is only synthesized by granule bound starch synthase, and the amylopectin is mainly synthesized by soluble starch synthase, starch branching enzyme (SBE), and debranching enzyme. The SBE in cereal crops has three isoforms: SBEI, SBEIIa, and SBEIIb, and is responsible for the formation of branch points of amylopectin [1, 2]. Suppressing or eliminating one or more SBE activities in rice, maize, wheat, and barley can significantly change amylopectin structure including of the decrease of branching degree and the elongation of branch-chain length, and increase the content of resistant starch (RS), which cannot be digested in the upper gastrointestinal tract but functions as a substrate for bacterial fermentation in the large intestine [3–9]. Many high RS crops with long branch-chains of amylopectin have been developed via mutation of SBE gene or inhibition of SBE expression. Their starches have high resistance to digestion and their derived food products can lower the glycemic and insulin responses and reduce the risk of developing type II diabetes, obesity, and cardiovascular disease [3–9].

For plants, seeds provide energy for germination and seedling growth. Starch is the main storage compound in cereal endosperm and serves as the primary source of carbohydrate during germination and seedling growth. Recently, Shaik et al. [10] report that starch bioengineering affects biomass remobilization during germination and seedling establishment, most probably through the combination of direct effects on the starch granule and molecular structure and the indirect effects on amylase activities. Maize *sbeI* mutant starch has an altered branching pattern for amylopectin and amylose. When *sbeI* mutant kernels are germinated, the mutant is associated with shorter coleoptile length and higher residual starch content, indicating that less efficient starch utilization impairs seedling growth [11]. However, a rice mutant RS4, which has about 10% RS content in the milled cooked rice, exhibits similar seed vigour to its wild type rice, indicating that RS has no negative impact on seed vigour, although RS cannot be hydrolyzed by α-amylase from human and animal in vitro [12].

A rice line (TRS) with high RS has been developed from an *indica* rice cultivar Te-qing (TQ) in our laboratory [7]. TRS endosperm contains 14.6% RS, and has shown significant potential to improve the health of the large intestine in rats [7]. Four differently morphological (heterogeneous) and regionally distributed starch granules have been identified in TRS mature kernel. They are polygonal, aggregate, elongated, and hollow starch granules, and distributed in endosperm cells from the inner to the outer of kernel [13]. These heterogeneous starch granules have significantly different structure and properties [14], resulting in different hydrolysis patterns by amylase [15]. The polygonal and aggregate starches have monophasic degradation patterns during hydrolysis, and the aggregate starch has higher resistance to hydrolysis than the polygonal starch. However, the elongated and hollow starches are rapidly degraded at the initial stage of hydrolysis but have high resistance to degradation in the following period, leading to a biphasic hydrolysis pattern [15].

In this study, TQ and its derived high RS rice TRS were used to investigate the seedling growth, starch property changes, and starch in situ degradation during seedling growth. Our objective was to further understand whether high RS starch influenced the dynamics of starch in situ degradation in seed and the seedling growth.

## Methods

### Plant materials

The high RS rice line TRS was generated from an *indica* rice cultivar Te-qing (TQ) after inhibition of both SBEI and SBEIIb through an antisense RNA technique [7]. Mature seeds were used to prepare the germinating seedlings.

### Preparation of germinating seedling

The rice seeds were imbibed in distilled water (the water was changed three times every day) in the dark at 28 °C for 2 days. The germinated seeds were placed in a well of a 96-well plate with embryo up. The lower two-third of seed was soaked in water (the water was changed every day) in the dark at 28 °C for seedling growth. Samples were taken out at 1, 4, 8, 12 and 16 day after imbibition (DAI).

### Measurement of the height of shoot and the dry weight of shoot and root

The shoot height was measured from the culm base to the tip of the longest leaf. The shoot and root were separated from the seedling and dried in oven at 110 °C for 3 h and at 80 °C for 2 d. Their dry weight was measured.

### Determination of the weight, total starch content, and soluble sugar content of germinated seed

The seeds without embryo, shoot and root were freeze-dried and weighted. The dry seeds were extensively ground into flour. The soluble sugar was extracted from flour in 80% (*v*/v) ethanol for 30 min at 80 °C and then centrifuged at 5000 *g* for 10 min. The resulting pellet was further washed two times with 80% ethanol. The combined supernatants were measured for soluble sugar by the colorimetric method of anthrone-H_2_SO_4_ [16]. The ethanol-insoluble residue was used for total starch content determination by using Megazyme Total Starch Assay Kit (K-TSTA).

### In vitro culture of mature embryos

Dehulled seeds were washed with distilled water and sterilized with 70% ethanol for 45 s, followed by shaking in a 2% sodium hypochlorite for 30 min. After that, the seeds were rinsed with sterile distilled water five times and dried. The embryos were carefully excised aseptically from the surrounding endosperm and placed in plastic tissue culture bottle (300 mL) of 10 mm depth and 7 mm diameter containing 30 mL normal MS medium. The medium was divided into two parts, thus allowing ten TQ and TRS embryos to grow separately in one bottle. The cultured embryos were incubated under 12-h photoperiod at 28 °C. After 6 days, the seedlings were photographed, and their heights were measured.

### Isolation of starch

The germinated seeds without embryos were homogenized in a mortar and pestle with H_2_O. The homogenate was filtered with 100-, 200-, and 400-mesh sieves, successively. The starch was washed three times with H_2_O and two times with anhydrous ethanol, freeze-dried, ground into powder, and passed through a 100-mesh sieve.

### Analysis of starch molecular weight distribution

The protein was removed from isolated starch with protease and sodium bisulfite, and debranched with isoamylase following the modified procedures of Lin et al. [17] according to the methods of Tran et al. [18] and Li et al. [19]. The debranched starch was analyzed using gel permeation chromatography (GPC) system (PL-GPC 220, Agilent Technologies UK limited, UK) with three columns (PL110–6100, 6300, 6525) and a differential refractive index detector.

### Analysis of starch crystalline structure

The crystalline structure of isolated starch was analyzed using an X-ray powder diffractometer (XRD) (D8, Bruker, Germany). The sample was exposed to the X-ray beam at 40 mA and 40 kV, and scanned from 3 to 40° 2θ with a step size of 0.02° [20].

### Analysis of starch short-range ordered structure

The short-range ordered structure of isolated starch was analyzed using a Varian 7000 Fourier transform infrared (FTIR) spectrometer with a DTGS detector equipped with an attenuated total reflectance single reflectance cell containing a germanium crystal (45° incidence-angle) (PIKE Technologies, USA). The original spectrum was corrected by subtraction of the baseline in the region from 1200 to 800 cm^−1^ before deconvolution was applied using Resolutions Pro. The assumed line shape was Lorentzian with a half-width of 19 cm^−1^ and a resolution enhancement factor of 1.9 [20].

### Histochemical analysis of starch in situ degradation in seed

The germinated seeds were first fixed in a 2.5% glutaraldehyde fixation solution in 0.1 M Na-phosphate butter for 2 h at room temperature and then held at 4 °C. The seeds were washed 3 times with phosphate buffer, successively dehydrated in gradient ethanol, and embedded in LR White Resin. The semithin sections of 2 μm thickness were cut with a glass knife on a Leica Ultrathin Microtome (EM UC7) following the method of Zhao et al. [21]. The sections were counterstained with periodic acid-Schiff reagent and toluidine blue O, and viewed with an Olympus BX53 light microscope equipped with a CCD camera.

### Statistical analysis

The one-way ANOVA with Tukey’s test, Pearson’s bivariate correlations, and Student’s *t* test were evaluated using the SPSS 16.0 Statistical Software Program.

## Results and discussion

### Seedling growth

**Fig. 1:**
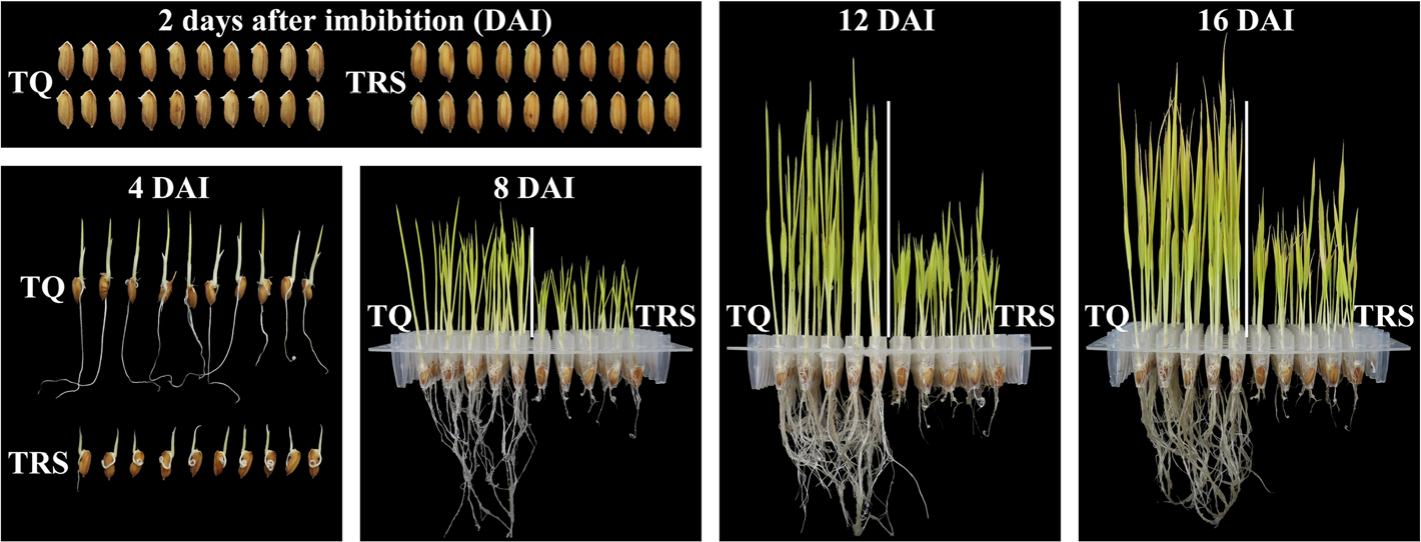
Photographs of rice seedlings

**Fig. 2:**
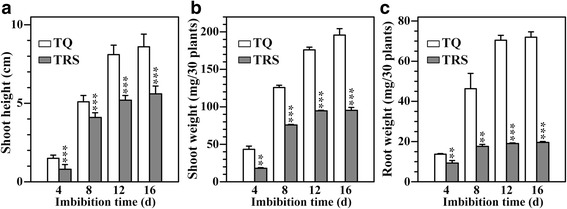
Shoot height (**a**) and the dry weight of shoot (**b**) and root (**c**) on 30 seeds basis during seedling growth. Values are means ± SD from 90 seedlings for (**a**) and three replicates for (**b** and **c**). Asterisks (*) highlight significant differences between TQ and TRS by Student’s *t* test (**P < 0.01; ***P < 0.001)

In order to investigate the influence of starch degradation on the seedling growth, the seeds were germinated and grew in the dark only in ddH_2_O. Under this growing condition, the only opportunity of the seed and germinating plantlet system to obtain energy is through respiration of storage compounds in seed [10]. The seed germination and seedling growth are shown in Fig. 1. The seeds began to germinate at about 2 DAI, but TRS germinated slightly slower than TQ. The seedling growth of TRS was significantly slower than that of TQ, indicating that the repression of SBE expression could be negatively correlated to seedling growth. The shoot height, shoot weight, and root weight were further investigated to quantitatively measure the effect of starch hydrolysis on seedling growth. Compared with TQ, the shoot height of TRS was significantly inhibited (Fig. 2a). The dry weight of shoot and root estimated on the 30 seeds basis (Fig. 2b and c) and on the same weight basis of pre-germinated seeds (Additional file 1: Figure S1) was significantly lower in TRS than in TQ, especially root weight.

**Fig. 3:**
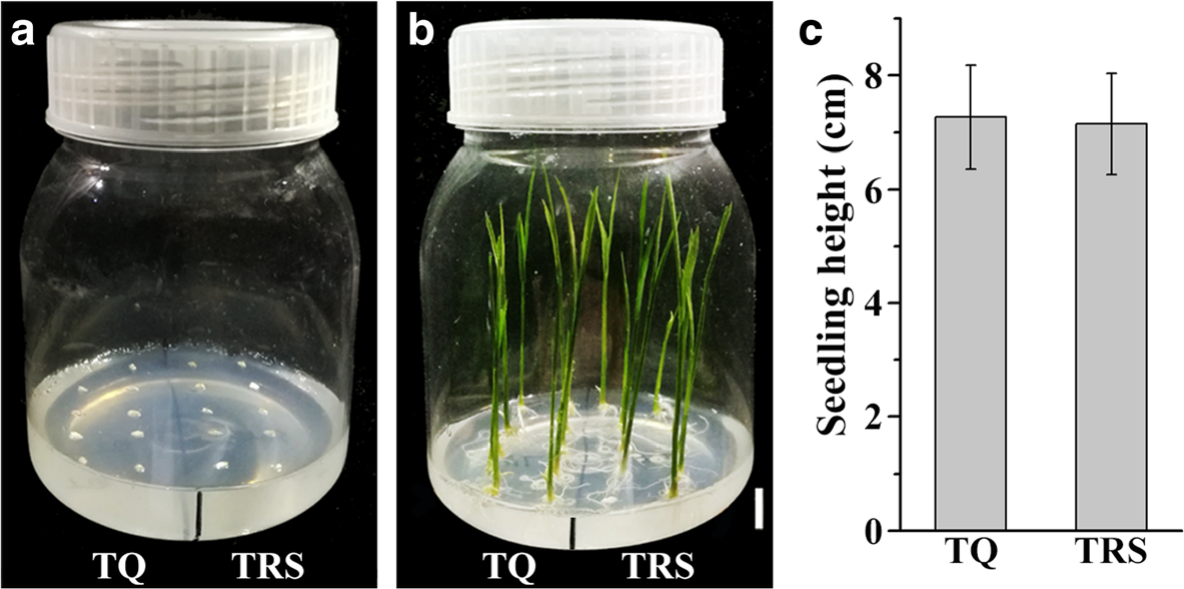
In vitro culture of mature embryo. (**a**), the excised embryos from TQ and TRS mature seeds were separately placed in normal MS medium; (**b**), seedlings at 6 days after in vitro culture, the scale bar = 1 cm; (**c**), the shoot height at 6 days after culture, values are means ± SD from 30 seedlings

In order to exclude the influence of embryo size and vitality on grain germination and seedling growth, the mature embryos were excised from the surrounding endosperm and in vitro cultured in normal MS medium under 12-h photoperiod at 28 °C. The seed germination and seedling growth did not show significant difference between TQ and TRS (Fig. 3). This result further showed that the slow growth of seedling resulted from the repression of SBE expression in endosperm and was not due to the embryo size and vitality.

### The material degradation of seed during seedling growth

**Fig. 4:**
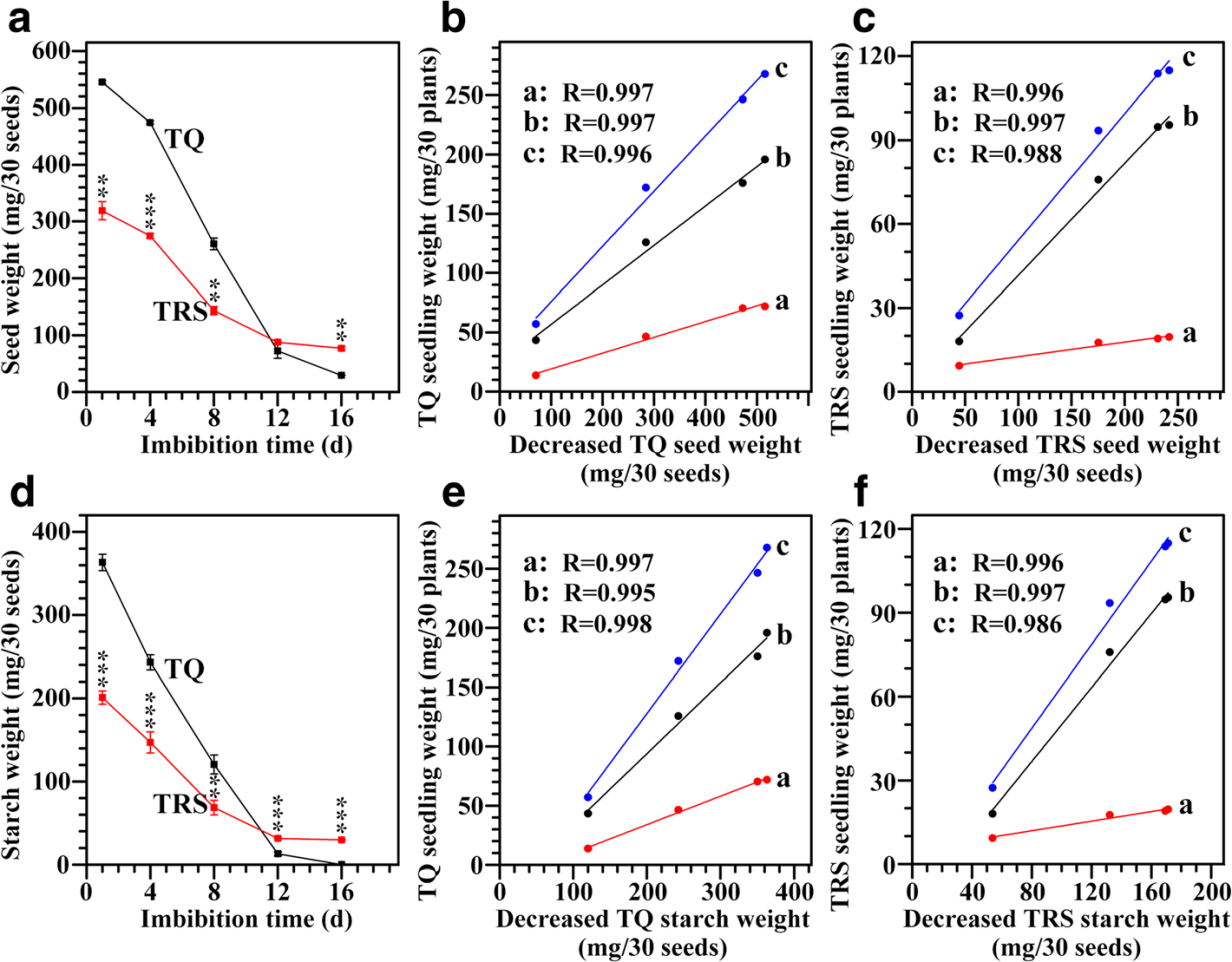
Seed and starch weights and their relationships with seedling weight on 30 seeds basis during seedling growth. (**a**), dry weight of seed without embryo; (**b**, **c**), the relationships between the decreased seed weight and the root (**a**), shoot (**b**), and seedling (root + shoot) weight (**c**) in TQ (**b**) and TRS (**c**); (**d**), dry weight of starch in endosperm; (**e**, **f**), the relationships between the decreased starch weight and the root (**a**), shoot (**b**), and seedling (soot + shoot) weight (**c**) in TQ (**e**) and TRS (**f**). For (**a** and **d**), values are means ± SD from three replicates, and asterisks (*) highlight significant differences between TQ and TRS by Student’s *t* test (**P < 0.01; ***P < 0.001). For (**b**, **c**, **e**, and **f**), values are the means of three replicates, and R indicates the regression coefficient

Grain serves as storage material for seed germination and seedling growth. During seedling growth, the weight of remaining seeds without embryos was measured (Fig. 4a). Before seed germination, TQ had significantly higher seed dry weight than TRS. After germination, the seed weight decreased faster in TQ than in TRS, and was significantly lower in TQ than in TRS at 16 DAI, indicating more mass was remobilized from TQ seeds than from TRS seeds. Shaik et al. [10] also reported that less dry mass was remobilized from the grains of the AO barley line (SBE RNAi suppressor) than from grains of a control wild type barley cultivar. The original biomass of seed had been remobilized to the emerging parts of seedling, such as root and shoot. In this study, we investigated the relationship between the decreased seed weight and the seedling weight (Fig. 4b and c). The decreased seed weight had significantly positive relations with the growth of root, shoot, and seedling (root + shoot) during seedling growth. Their correlation coefficients were 0.997, 0.997, and 0.996 for TQ, and 0.996, 0.997, and 0.988 for TRS, indicating that the slow growth of TRS seedling resulted from the slow degradation of seed material.

Starch is the main source of carbon during germination and seedling growth when seed was growth in the dark with water but no nutrients [10]. The starch weight in remaining seeds was also measured (Fig. 4d). The starch in TQ seeds was rapidly degraded at a similar rate before 12 DAI, but the degradation of starch in TRS seeds was slow before 12 DAI and remained high resistance to hydrolysis. The relationship between the decreased starch weight and the seedling growth was also investigated in this study (Fig. 4e and f). A similar effect was confirmed that the decreased starch weight was significantly positively related with the weight of root, shoot, and seedling (root + shoot) during seedling growth. Their correlation coefficients were 0.997, 0.995, and 0.998 for TQ, and 0.996, 0.997, and 0.986 for TRS, indicating that the slow growth of TRS seedling resulted from the slow hydrolysis of seed starch. Our results were in agreement with the report of Shaik et al. [10]. The impeded remobilization of biomass from TRS seeds to plantlets resulted in the slow growth of TRS seedling. It was previously shown that TRS starch isolated from seed has a higher resistance to enzyme hydrolysis than TQ starch [8]. The above results showed that the reduced biomass remobilization in TRS grains was due to a higher fraction of undegraded starch, which was confirmed by residual starch content in seeds at 12 and 16 DAI.

Considering that TRS had a much lower seed weight and starch content than TQ (Fig. 4a and d), it was possible that the slow material degradation of TRS seed was due to the low seed weight and starch weight. The data of the material degradation of seed and the dry weight of seedling were re-estimated on the same weight basis of pre-germinated seed (Additional file 2: Figure S2). Though the weight of seed and starch was linearly decreased and did not show significant difference between TQ and TRS before 8 DAI, the weight of residual seed and starch was significantly higher in TRS than in TQ after 8 DAI, indicating that residual starch in TRS seed was highly resistant to in situ degradation. Similar results have been reported in barley AO line: it efficiently degrades starch initially but shows restricted starch degradation at mid and late stages of seedling growth [10].

### Variation of soluble sugar content in endosperm during seedling growth

**Fig. 5:**
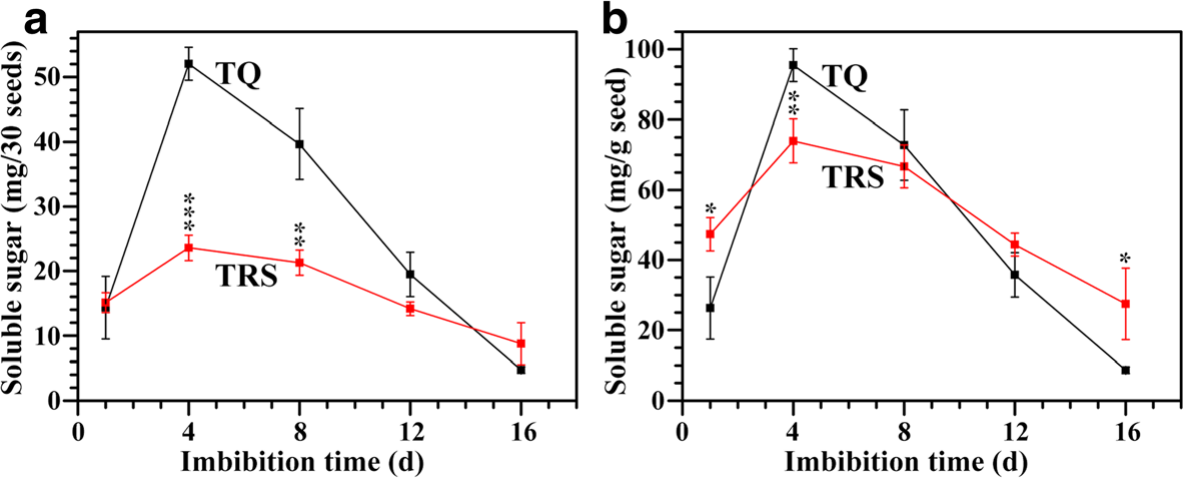
The soluble sugar content of endosperm on 30 seeds basis (**a**) and on the weight basis of pre-germinated seeds (**b**) during seedling growth. Values are means ± SD from three replicates, and asterisks (*) highlight significant differences between TQ and TRS by Student’s *t* test (*P < 0.05; **P < 0.01; ***P < 0.001)

Seedling growth is directly supported by soluble sugar. Given the changes in the starch content as carbon sources, the total soluble sugar in remaining endosperm was determined during germination and seedling growth. The soluble sugar content in TRS seed was similar to that in TQ seed at 1 DAI, but the significant difference in soluble sugar content appeared at 4, 8, and 16 DAI. The soluble sugar content in TQ seed was significantly higher than in TRS seed at 4 and 8 DAI, but lower at 16 DAI (Fig. 5a). The soluble sugar variation was also determined on the same weight basis of pre-germinated seed (Fig. 5b). The soluble sugar content was significantly higher in TRS than in TQ at 1 DAI, but TQ seed contained significantly higher soluble sugar than TRS seed at 4 DAI, indicating that TQ starch was degraded faster than TRS starch from 1 DAI to 4 DAI. The soluble sugar content was significantly higher in TRS than in TQ at 16 DAI, which was due to that the starch in TQ endosperm was completely degraded at 16 DAI. The soluble sugar change in remaining endosperm during seedling growth agreed with the starch degradation in seed on both 30 seeds basis and on weight basis of pre-germination seeds.

### Variation of starch components during seedling growth

**Fig. 6:**
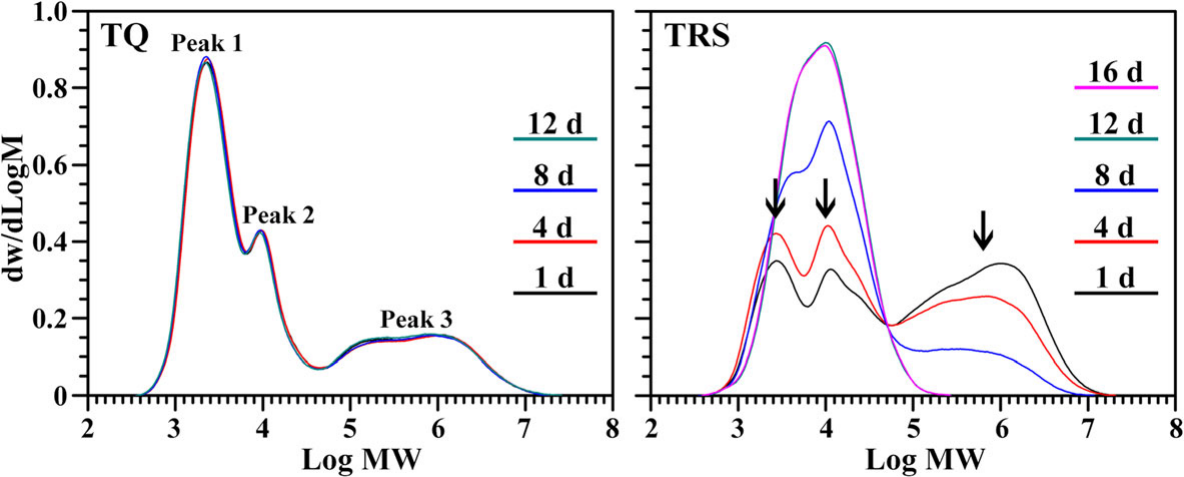
GPC chromatogram of isoamylase-debranched starch isolated from endosperm at different days after imbibition. The arrows indicate the main differences in GPC chromatogram between TQ and TRS

Starch is mainly composed of both amylose and amylopectin, and the amylopectin contains short branch-chains (A and short B chains) and long branch-chains (long B chains). The proportions of amylose, amylopectin short branch-chains, and amylopectin long branch-chains in starch can be determined using GPC of isoamylase-debranched starch [22]. In the present study, the starches were isolated from seeds during seedling growth, and debranched using isoamylase. Their GPC chromatograms are shown in Fig. 6. Usually, the GPC chromatogram of isoamylase-debranched starch has typical three peaks, dominated Peak 1, Peak 2 and Peak 3, presenting the amylopectin short branch-chains, amylopectin long branch-chains, and amylose, respectively. Their area percentages show their component proportion in starch, and the area ratio of Peak 1 to Peak 2 reflects the relative content of short and long branch-chains in amylopectin and is negatively correlated with the amylopectin long branch-chains [22, 23]. The GPC profiles and component content of TQ seed starch did not significantly change during seedling growth, indicating that the components of starch were simultaneously degraded (Fig. 6, Table 1). However, the GPC chromatograms of TRS seed starches showed significant difference during seedling growth (Fig. 6). The amylose content decreased fast from 53.4% at 1 DAI to 41.7% at 4 DAI and 15.0% at 8 DAI. At 12 DAI, amylose in TRS seed was not detected, indicating that amylose in TRS seed starch was degraded faster than amylopectin during seedling growth. Shaik et al. [10] measured the optical density 620 nm/550 nm (OD620/550) to assess the relative content of amylose and amylopectin during barley seedling growth. For wild type barley, the OD620/550 was constant throughout the entire course of seedling growth while for the AO line there was a decline in this ratio, indicating that neither amylose nor amylopectin was preferably degraded in wild type barley but amylose was preferably degraded compared to amylopectin in AO line. In addition, the constant area ratio of Peak 1/Peak 2 during TQ seedling growth also indicated that the short and long branch-chains of amylopectin were synchronously degraded, but the gradually decline of the ratio during TRS seedling growth indicated that the long branch-chains of amylopectin were more resistant to hydrolysis than the short branch-chains (Table 1).

**Table 1:** Molecular weight distribution of starch isolated from endosperm during seedling growth^a^

Day after imbibition	Peak area of GPC of starch from TQ endosperm				Peak area of GPC of starch from TRS endosperm			
	Peak 1 (%)	Peak 2 (%)	Peak 3 (%)	Peak 1/Peak 2	Peak 1 (%)	Peak 2 (%)	Peak 3 (%)	Peak 1/Peak 2
1 d	52.6 ± 0.7a	20.3 ± 0.2a	27.2 ± 0.5a	2.60 ± 0.06a	21.9 ± 0.2a	24.7 ± 0.3a	53.4 ± 0.5c	0.89 ± 0.00c
4 d	54.2 ± 0.4a	19.6 ± 0.2a	26.2 ± 0.2a	2.76 ± 0.05a	26.0 ± 0.4b	32.4 ± 0.3b	41.7 ± 0.8b	0.80 ± 0.01b
8 d	53.4 ± 0.5a	19.8 ± 0.3a	26.8 ± 0.8a	2.69 ± 0.02a	30.9 ± 0.5c	54.1 ± 0.1c	15.0 ± 0.4a	0.57 ± 0.01a
12 d	52.8 ± 0.3a	19.8 ± 0.5a	27.4 ± 0.2a	2.66 ± 0.08a	34.0 ± 0.6c	66.0 ± 0.6d	–	0.51 ± 0.01a
16 d	–	–	–	–	32.7 ± 2.4c	67.3 ± 2.4d	–	0.49 ± 0.05a

^a^Data are means ± standard deviations, *n* = 2. Values in the same column with different letters are significantly different (*P* < 0.05)

^–^Data are not detected

### Variation of crystalline structure of starch during seedling growth

**Fig. 7:**
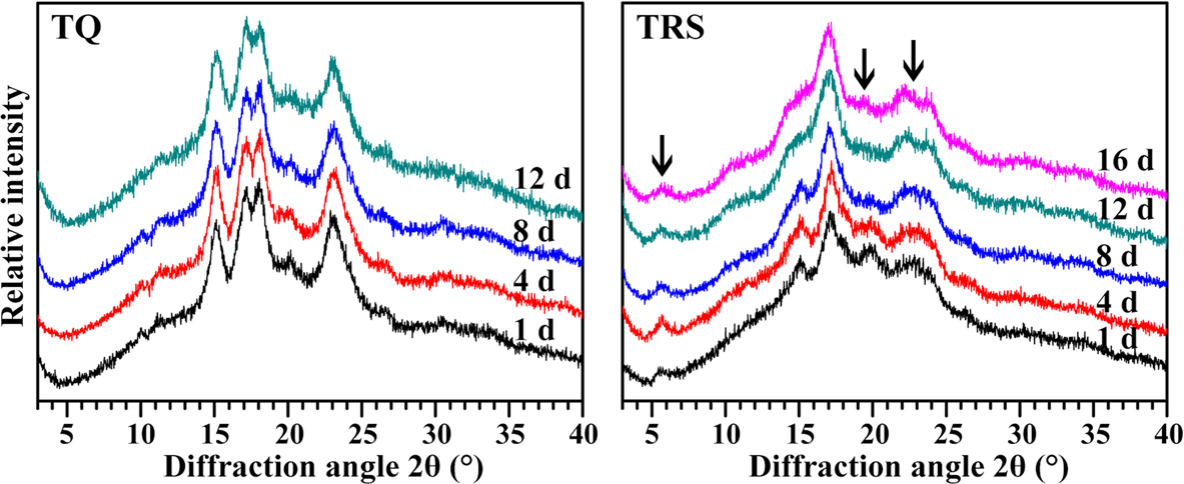
XRD pattern of starch isolated from endosperm at different days after imbibition. The arrows indicate the main differences in XRD pattern between TQ and TRS

Starches are classified into A-, B-, and C-type according to their XRD patterns. A-type starch has strong diffraction peaks at about 15° and 23° 2θ, and an unresolved doublet at around 17° and 18° 2θ. B-type starch shows strong peak at 17° 2θ, a few small peaks at around 15°, 20°, 22°, and 24° 2θ, and a characteristic peak at about 5.6° 2θ. Typical C-type starch has strong diffraction peaks at about 17° and 23° 2θ, and a few small peaks at around 5.6° and 15° 2θ [24]. A- and B-type starch contains only A-type and B-type crystallinity, respectively. However, C-type starch is a mixture of A- and B-type crystallinity [25]. Amylopectin with short branch-chains and closed branching points can favorably form A-type crystallinity, and amylopectin with long branch-chains and distant branching points does B-type crystallinity [26]. The XRD patterns of starches from TQ and TRS seeds during seedling growth are presented in Fig. 7. The starches from TQ seeds during seedling growth had similar XRD patterns, and showed typical A-type crystallinity. However, the starches from TRS seeds during seedling growth had significantly different XRD patterns. The starch from TRS seeds of 1 DAI showed typical C-type XRD pattern. In addition, a slight shoulder peak at about 18° 2θ, which is indicative of the A-type polymorph, was also observed, indicating that this starch contained higher proportion of A-type crystallinity than B-type crystallinity. With the time increase of TRS seed imbibition, the shoulder peak at about 18° 2θ quickly vanished (about 4 DAI), and the peak at about 23° 2θ gradually became wide (8 DAI) and was divided into two peaks at about 22° and 24° 2θ (16 DAI), which are indicative of the B-type polymorph. The spectrum change of TRS seed starch indicated that the A-type crystallinity was preferentially degraded or degraded faster than the B-type crystallinity during seedling growth. It was also noteworthy that the intensity of peak at about 20° 2θ, which was an amylose-lipid complex diffraction peak, quickly decreased during seedling growth. The above results indicated that the B-type crystallinity in TRS starch was highly resistant to in situ degradation during seedling development, and agreed with the in vivo and in vitro digestion of isolated TQ and TRS starch [8]. The crystalline variation of TQ and TRS seed starch during seedling growth also agreed with the results of GPC that the long branch-chains in amylopectin, which is prone to form B-type crystallinity, was more resistant to in situ hydrolysis than the short branch-chains.

### Variation of short-range ordered structure of starch during seedling growth

**Fig. 8:**
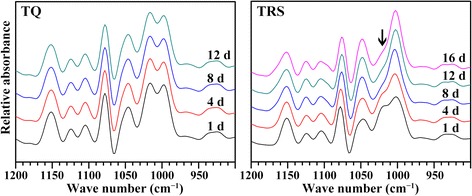
FTIR pattern of starch isolated from endosperm at different days after imbibition. The arrows indicate the main differences in FTIR spectrum between TQ and TRS

Starch contains amorphous and ordered structure. The amorphous structure is consisted of amylose, and the ordered structure composes of two types of helices from amylopectin branch-chains: the short-range ordered structure, defined as the double-helical order, and the long-range ordered structure, defined as the ordered crystallinity packed by double helices. The former is sensitive to the infrared spectrum, and the latter can be detected by XRD [27]. For infrared spectrum, the band at 1045 cm^−1^ is linked with order/crystalline regions in starch, the band at 1022 cm^−1^ arises as a result of absorption by stretching modes in amorphous starch and is sensitive to amorphous structure, and the band at 995 cm^−1^ results from bonding in hydrated carbohydrate helices [28]. The FTIR spectra of starches during rice seedling growth are presented in Fig. 8. For TQ, the FTIR spectrum did not significantly change during seedling growth. Whereas for TRS starch, the FTIR spectrum was similar except that the 1022 cm^−1^ absorbance band gradually decreased during rice seedling growth. The results indicated that the ordered and amorphous structures were simultaneously degraded in TQ starch, but the amorphous structure was degraded faster than the crystalline structure in TRS starch. The result was in agreement with the in vivo and in vitro digestion of isolated TQ and TRS starch [8], indicating that the amylopectin, not the amylose, was resistant to enzymatic hydrolysis.

### In situ degradation of starch granule in seed during seedling growth

**Fig. 9:**
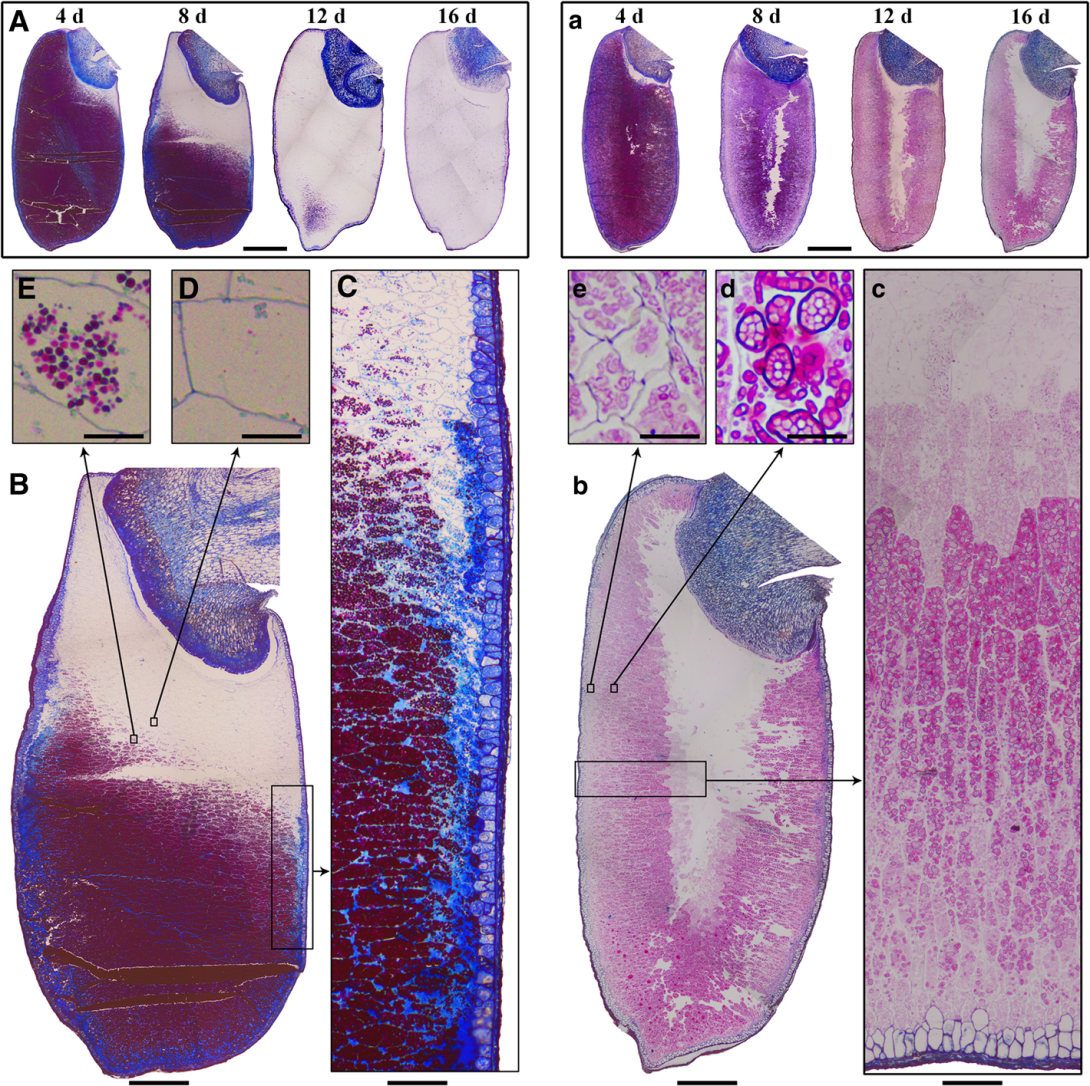
In situ degradation of starch granule in endosperm at different days after imbibition. (**A**-**E**): TQ seed; (**B**-**E**): the magnification of germinated seeds at 8 DAI; (**a**-**e**): TRS seed; (**b**-**e**): the magnification of germinated seeds at 16 DAI. The whole seed longitudinal section was counterstained with periodic acid-Schiff’s and toluidine blue O. Scale bar = 1 mm (**A**, **a**), 500 μm (**B**, **b**), 100 μm (**C**, **c**), and 20 μm (**D**, **E**, **d**, **e**)

The in situ degradation of starch in endosperm during seedling growth was investigated using our established method for preparing the whole section of mature cereal seed [21]. The longitudinal sections of whole seeds at 4, 8, 12, and 16 DAI were counterstained with periodic acid-Schiff reagent to visualize the starch granules (red) and with toluidine blue O to show the histological structure (blue) (Fig. 9). For TQ, starch was gradually degraded from the proximal to distal region of embryo and from the outer to inner of endosperm (Fig. 9A). At 8 DAI, the starch in the endosperm close to aleurone layer was partly degraded (Fig. 9C), and that close to embryo was completely degraded (Fig. 9D). These results agreed with that embryo and aleurone layer secrete the amylase to degrade starch in the endosperm [10]. At 12 DAI, the starch was completely degraded except the distal region of embryo (Fig. 9A). The degraded pattern of starch in TQ endosperm during seedling growth was in agreement with that of other cereal crops [10, 12].

In TRS seed, the starch degradation was complex. The starch in the endosperm close to aleurone layer and embryo was first partly degraded, then the starch in the inner region of endosperm was completely degraded, and the starch in the outer and middle regions of endosperm, especially in the middle region, could not be completely degraded even at 16 DAI (Fig. 9a). At 16 DAI, the inner region of endosperm was devoid of starch, but the middle and outer regions of endosperm were stained red, indicating the existence of starch residual (Fig. 9b). TRS endosperm contained polygonal, aggregate, elongated, and hollow starch granules. They were specifically distributed in different regions from the inner to outer of kernel [13]. The polygonal starch granules in the inner region of endosperm are subgranules of compound starch and have A-type crystallinity, are similar to TQ starch in morphology, molecular component, and structural properties [14]. Therefore, it was easy to understand for its complete degradation at 16 DAI. The aggregate starch in the middle of endosperm is semicompound C-type granules, and consisted of packed smaller subgranules and a thick band encircling the entire circumference of the granules [29, 30]. The A-type crystallinity is located in the central region of subgranule and can be hydrolyzed by amylase, and the B-type crystallinity is located in the peripheral region of the subgranules and the surrounding band of the starch granule and shows very high resistance to amylase hydrolysis [8, 29]. The elongated starch is also the aggregate granule of subgranules in linear arrangement, has C-type crystallinity, and shows high resistance to in vitro digestion [8, 15, 29]. Therefore, the middle region of TRS endosperm, in which aggregate and elongated starches are mainly distributed, had the starch residual in the peripheral region of the subgranules and the surrounding band of aggregate and elongated starches at 16 DAI (Fig. 9d). The outer region of TRS endosperm, in which hollow starch is distributed, was first easy degraded for the high content of amorphous starch. But the residual of hollow starch was detected at 16 DAI (Fig. 9e), indicating that hollow starch had high resistance to hydrolysis. Huang et al. [15] also found the hollow starch is rapidly degraded at the early hydrolysis stage, but has high resistance to in vitro digestion for the existence of B-type crystallinity and high amylopectin long branch-chains. In addition, the starch residual at 16 DAI was also in agreement with the results of XRD that starch had B-type crystallinity.

## Conclusion

The starch in TQ seed was gradually degraded from the proximal to distal region of embryo and from the outer to inner of endosperm during seedling growth, and its components of amylose and amylopectin short and long branch-chains were simultaneously degraded. However, in TRS seed, the inner region of endosperm was completely degraded, but the middle and outer regions of endosperm was partly degraded during seedling growth. The B-type crystallinity and long branch-chains of amylopectin in TRS aggregate, elongated and hollow starches had high resistance to in situ degradation, which inhibited TRS seedling growth.

## Additional files

Additional file 1: Figure S1. Dry weight of shoot (A) and root (B) on the weight basis of pre-germinated seeds during seedling growth. Values are means ± SD from three replicates. Asterisks (*) highlight significant differences between TQ and TRS by Student’s *t* test (*P < 0.05; **P < 0.01; ***P < 0.001). (TIFF 2675 kb)

Additional file 2: Figure S2. Seed and starch weights and their relationships with seedling weight on the weight basis of pre-germinated seeds during seedling growth. (A), dry weight of seed without embryo; (B, C), the relationships between the decreased seed weight and the root (a), shoot (b), and seedling (root + shoot) weight (c) in TQ (B) and TRS (c); (d), dry weight of starch in endosperm; (e, f), the relationships between the decreased starch weight and the root (a), shoot (b), and seedling (soot + shoot) weight (c) in TQ (E) and TRS (F). For (a and d), values are means ± SD from three replicates, and asterisks (*) highlight significant differences between TQ and TRS by Student’s *t* test (*P < 0.05; **P < 0.01; ***P < 0.001). For (b, c, e, and f), values are the means of three replicates, and R indicates the regression coefficient. (TIFF 8768 kb)

## Abbreviations

DAI: Day after imbibition
FTIR: Fourier transform infrared
GPC: Gel permeation chromatography
RS: Resistant starch
SBE: Starch branching enzyme
XRD: X-ray powder diffractometer
