# Supplementary figures and images for: Long branch-chains of amylopectin with B-type crystallinity in rice seed with inhibition of starch branching enzyme I and IIb resist in situ degradation and inhibit plant growth during seedling development: Degradation of rice starch with inhibition of SBEI/IIb during seedling development
Source: BMC Plant Biol. 2018 Jan 8;18:9. doi: 10.1186/s12870-017-1219-8 (PMC5759222; doi:10.1186/s12870-017-1219-8)

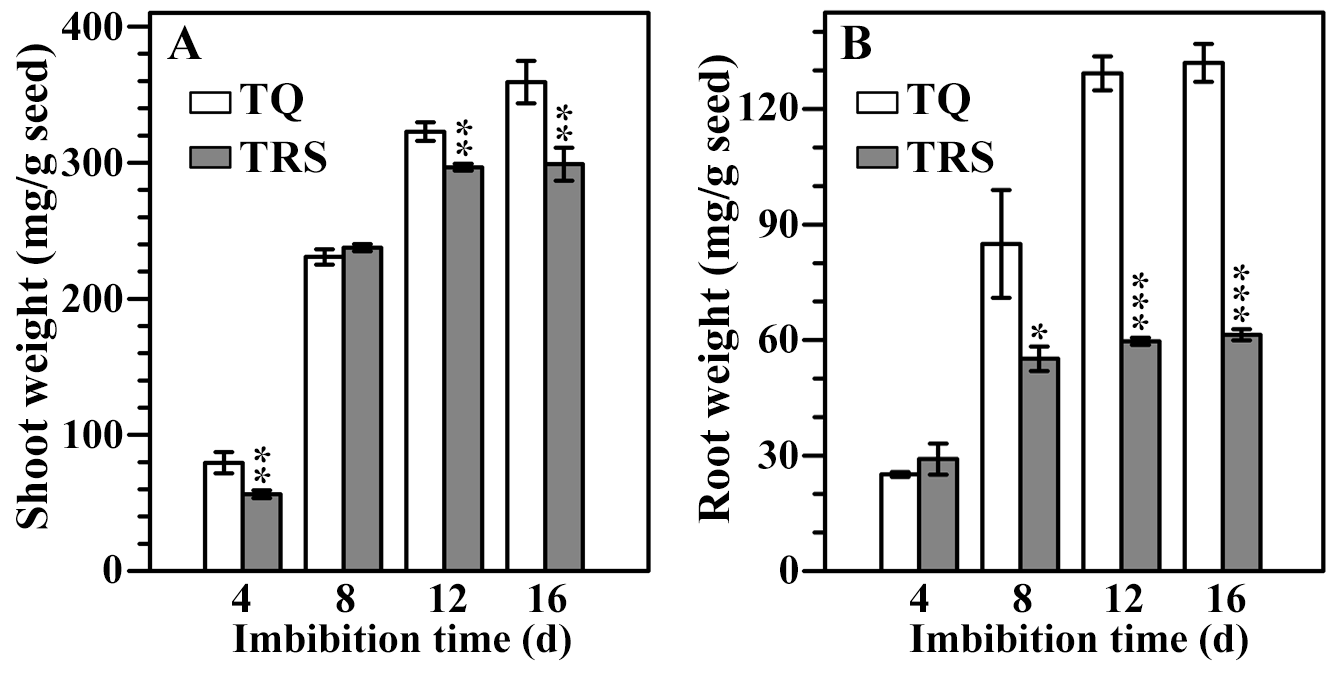

Supplement: Supplementary file 1 — Dry weight of shoot (A) and root (B) on the weight basis of pre-germinated seeds during seedling growth. Values are means ± SD from three replicates. Asterisks (*) highlight significant differences between TQ and TRS by Student’s t test (*P < 0.05; **P < 0.01; ***P < 0.001). (TIFF 2675 kb) [file 12870_2017_1219_MOESM1_ESM.tif]

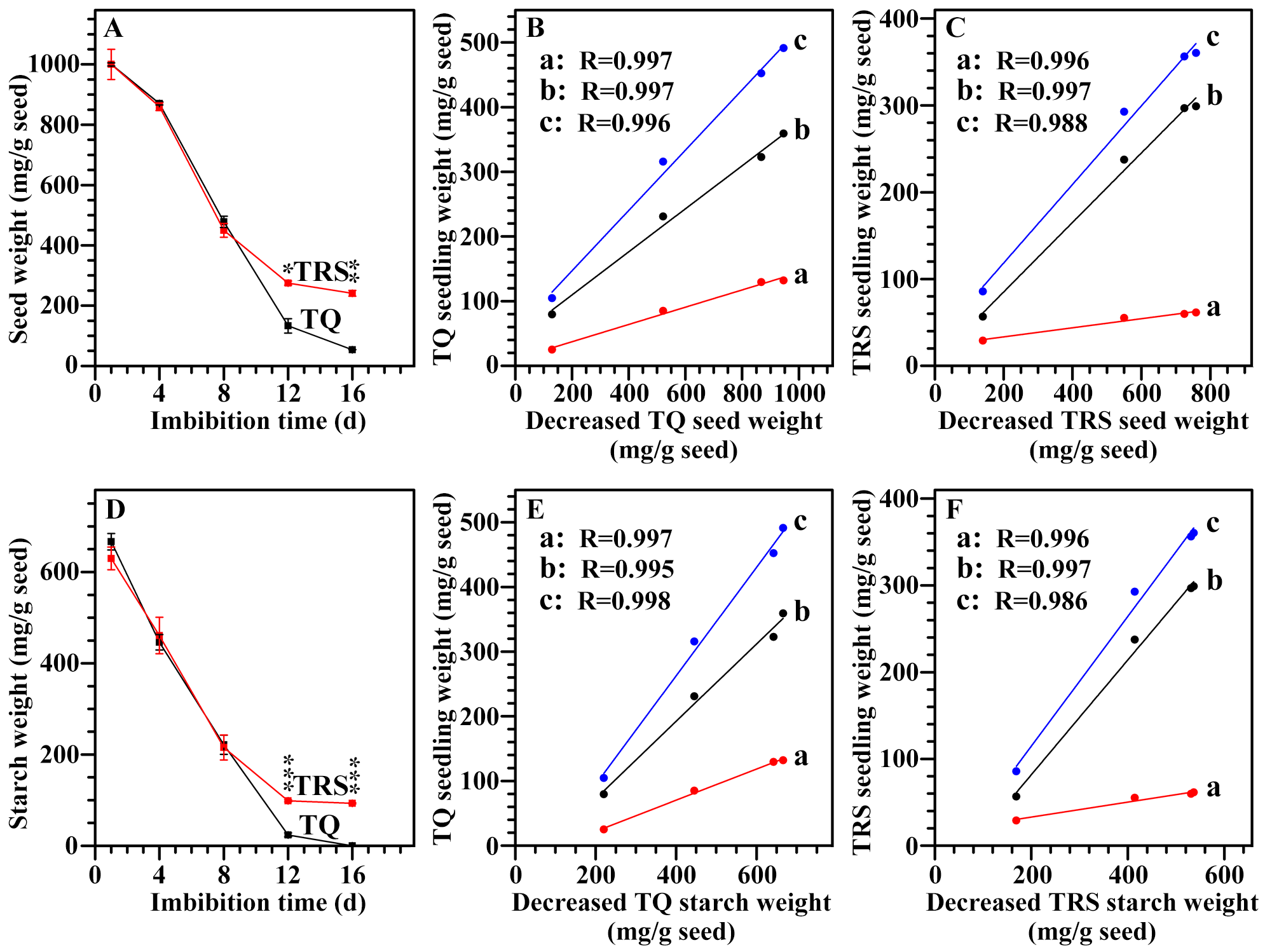

Supplement: Supplementary file 2 — Seed and starch weights and their relationships with seedling weight on the weight basis of pre-germinated seeds during seedling growth. (A), dry weight of seed without embryo; (B, C), the relationships between the decreased seed weight and the root (a), shoot (b), and seedling (root + shoot) weight (c) in TQ (B) and TRS (c); (d), dry weight of starch in endosperm; (e, f), the relationships between the decreased starch weight and the root (a), shoot (b), and seedling (soot + shoot) weight (c) in TQ (E) and TRS (F). For (a and d), values are means ± SD from three replicates, and asterisks (*) highlight significant differences between TQ and TRS by Student’s t test (*P < 0.05; **P < 0.01; ***P < 0.001). For (b, c, e, and f), values are the means of three replicates, and R indicates the regression coefficient. (TIFF 8768 kb) [file 12870_2017_1219_MOESM2_ESM.tif]
